# Self-controlled and low-frequency KP feedback enhances gymnastics skill learning via motivation and metacognition

**DOI:** 10.3389/fpsyg.2026.1769157

**Published:** 2026-04-23

**Authors:** Tianpeng Yang, Kai Li, Xinmiao Zhang, Yujun Cai

**Affiliations:** 1 School of Physical Education, Shanghai University of Sport, Shanghai, China; 2 College of Physical Education, Jiujiang University, Jiujiang, China; 3 College of Sports Industry and Leisure, Nanjing Sport Institute, Nanjing, China

**Keywords:** autonomous motivation, feedback frequency, metacognitive strategies, motor learning, self-controlled feedback, sequential mediation

## Abstract

**Background:**

Feedback plays a critical role in motor skill learning, yet the psychological and cognitive mechanisms through which feedback frequency and learner control influence long-term performance remain insufficiently understood. Theoretical models suggest that autonomy-supportive and spaced feedback may enhance motivation and metacognitive engagement, thereby promoting durable learning outcomes. This study investigated whether self-controlled and low-frequency knowledge of performance (KP) feedback enhances motor learning through a sequential motivational-cognitive pathway.

**Methods:**

A total of 232 novice university students practiced a goat stride vault (a multi-phase vaulting skill involving run-up, take-off, hand support, and controlled landing) under a 2 × 2 between-subjects design manipulating KP opportunity constraints (high availability vs. limited opportunities; two trials per 10-trial block) and feedback control (instructor-controlled vs. self-controlled). Autonomous motivation and metacognitive strategy use were assessed following acquisition. Performance during acquisition, retention, and transfer was evaluated. A mixed-design ANOVA tested performance changes across phases, and structural equation modeling examined a sequential motivational-cognitive mediation pathway.

**Results:**

Mixed ANOVA revealed a significant main effect of phase and significant phase × frequency, phase × control, and three-way interactions. SEM indicated significant indirect effects of feedback control and frequency on retention and transfer via autonomous motivation and metacognitive strategy use.

**Conclusion:**

Autonomy-supportive and spaced KP feedback enhances motor learning by strengthening autonomous motivation and metacognitive engagement. This motivational-cognitive mechanism provides evidence-based guidance for designing effective instructional feedback in complex motor-skill learning.

## 1 Introduction

Learning complex gymnastics skills involves transforming explicit technical cues into coordinated and fluid movement patterns performed under time and postural constraints (Magill and Anderson, 2010; Schmidt et al., 2018). In this process, augmented feedback—especially knowledge of performance (KP) delivered through video replay and targeted cues—supports technique refinement, movement stabilization, and skill consolidation (Kok et al., 2020; Mödinger et al., 2022). However, in applied instruction settings, two design questions remain central: how often feedback should be provided and who should determine its timing. Clarifying these issues is important for evidence-based coaching and for refining motor-learning theory.

### 1.1 Augmented feedback and frequency

Motor learning reflects relatively durable changes in skill capability rather than temporary performance fluctuations (Schmidt et al., 2018). Early work showed that frequent feedback can facilitate short-term acquisition performance, yet overly frequent external cues may undermine learners' internal error-detection and self-correction once feedback is withdrawn (Salmoni et al., 1984; Winstein and Schmidt, 1990). This pattern underlies the Guidance Hypothesis, which posits that reduced feedback can promote learning by encouraging reliance on intrinsic information sources (Winstein and Schmidt, 1990). Consistent with this view, reduced feedback frequency (e.g., providing KP on a subset of trials) often yields better retention and transfer than continuous feedback (Chiviacowsky and Wulf, 2002). The challenge-point framework further suggests that optimal feedback depends on task complexity and learner skill, rather than a simple “more is better” principle (Guadagnoli and Lee, 2004; Kantak and Winstein, 2012; Lee and Carnahan, 2021). With advances in high-speed cameras and instant video replay, video-based KP has become more ecologically valid and may support precise movement analysis in complex skill learning (Potdevin et al., 2018; Mödinger et al., 2022). Nevertheless, how feedback frequency interacts with learners' self-regulatory processes to shape durable learning remains incompletely understood (Efklides, 2011; St. Germain et al., 2023).

### 1.2 Autonomy support and self-controlled feedback

Beyond frequency, feedback control—whether feedback is determined by the instructor or the learner—is an influential instructional lever (McKay et al., 2022b). From a Self-Determination Theory (SDT) perspective, autonomy-supportive contexts satisfy basic psychological needs and enhance autonomous motivation and sustained engagement (Deci and Ryan, 2000; Ryan and Deci, 2000; Manninen et al., 2022). Self-controlled feedback is often considered autonomy supportive because learners decide whether/when to receive KP, potentially increasing perceived competence and ownership of learning (Sanli et al., 2013; Wulf and Lewthwaite, 2016; Bacelar et al., 2022).

From an information-processing standpoint, learners may request feedback after trials perceived as successful or uncertain, making each feedback instance more personally meaningful and potentially more informative for subsequent correction (Chiviacowsky and Wulf, 2002). Recent work has also begun to examine the timing aspect of self-controlled feedback. Akizuki et al. (2025) found that allowing learners to choose feedback timing improved error estimation and learning, whereas yoked controls did not show the same benefits (Carter and Ste-Marie, 2017). Together, these findings suggest that autonomy over when feedback is accessed may strengthen both motivational engagement and information processing. At the same time, benefits are not universal: meta-analytic evidence indicates that effects vary with task constraints, feedback type (KR vs. KP), and practice conditions (McKay et al., 2022a), and autonomy may impose additional demands under high cognitive load or limited self-regulation skills (Grand et al., 2015). Thus, identifying when and why self-controlled feedback is effective requires a clearer mechanistic account.

### 1.3 Metacognitive processes as a pathway from feedback to learning

Metacognition—planning, monitoring, and evaluating one's actions—may be a key cognitive mechanism by which feedback contributes to long-term learning. In motor learning, metacognitive control processes—deliberate monitoring, evaluation, and strategic adjustment—help convert informational inputs (including KP) into durable skill changes (Simon and Bjork, 2001; Efklides, 2011). Timely KP provides information for the metacognitive cycle, supporting reflective planning and error-based adjustment across trials (Efklides, 2011). When learners decide when to access KP, feedback may be integrated more effectively into monitoring and evaluation, strengthening self-regulated practice. In contrast, high-frequency externally controlled feedback may reduce metacognitive involvement by fostering reliance on external cues and diminishing active error estimation (Hewitson et al., 2023).

Motivation and metacognition are positively related but not interchangeable: motivation reflects willingness and persistence, whereas metacognition reflects strategic control of performance and learning (Zimmerman, 2002; Efklides, 2011). A plausible sequential account is that autonomy-supportive feedback first enhances autonomous motivation, which then increases metacognitive strategy use during practice, ultimately supporting retention and transfer. This sequential assumption is also consistent with self-regulated learning perspectives, which suggest that motivational readiness supports learners' willingness to engage in planning, monitoring, and evaluation during practice rather than these processes emerging independently of motivation (Zimmerman, 2002; Efklides, 2011). In this sense, autonomous motivation may function as an initiating condition for metacognitive engagement, providing a theoretical basis for testing a sequential rather than purely parallel mediation pathway. At the same time, because motivation and metacognition are closely related in applied learning contexts, alternative specifications should also be considered when interpreting the model. Despite this theoretical logic, few studies have modeled motivation and metacognition within a single mechanism-focused framework in complex, ecologically valid motor tasks.

### 1.4 The present study and hypotheses

Evidence on feedback frequency and learner autonomy is substantial but not unequivocal. Reduced feedback may prevent dependency and deepen processing, whereas higher-frequency feedback can facilitate early calibration in novices; similarly, self-controlled feedback may enhance engagement but depends on effective self-regulation and task constraints (McKay et al., 2022a,b). Accordingly, the present study tests frequency and control as complementary instructional levers and evaluates an a priori motivation-metacognition pathway while acknowledging these boundary conditions.

**Figure 1:**
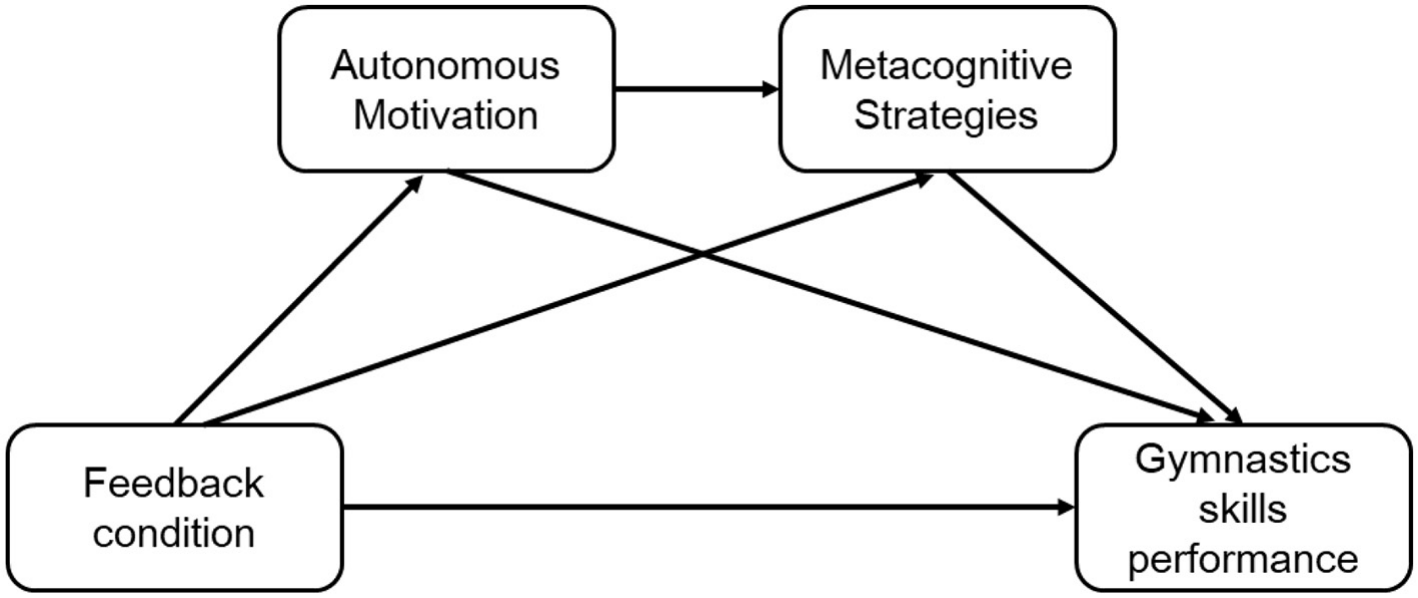
Hypothesized relationships among feedback, motivation, metacognitive strategies, and gymnastics skill learning. H5 was tested as an exploratory moderated mediation by comparing indirect effects across frequency conditions (multi-group SEM); moderation is not depicted in the conceptual diagram for simplicity.

To address the gap in mechanism-focused modeling in an ecologically valid gymnastics context, we used structural equation modeling (SEM) to test a theory-driven sequential mediation model examining how feedback frequency and feedback control influence autonomous motivation and metacognitive strategy use, and in turn, retention and transfer performance in a multi-phase vaulting task (Figure 1). The hypotheses were:

- **H1:** Feedback control (self- vs. instructor-controlled) and feedback frequency (low vs. high) are expected to be associated with retention and transfer performance, such that self-controlled and low-frequency feedback are linked to higher mean performance.
- **H2:** Feedback conditions will influence performance indirectly through autonomous motivation (single mediation).
- **H3:** Feedback conditions will influence performance indirectly through metacognitive strategy use (single mediation).
- **H4:** A sequential mediation pathway will emerge (motivation → metacognition), such that self-controlled feedback enhances motivation, which then increases metacognitive engagement and improves learning outcomes.
- **H5:** Indirect effects are expected to differ across feedback-frequency conditions (exploratory moderated mediation).

## 2 Methods

### 2.1 Participants

A total of 232 university students at the beginner level in gymnastics participated in the study (110 women, 122 men). All were enrolled at a comprehensive university in eastern China. The mean age was 19.23 years (SD = 2.70).

Inclusion criteria were: no prior systematic training in vaults over a box horse or similar tasks, and a valid medical certificate confirming that they could safely participate in physical activity. Exclusion criteria included current musculoskeletal injury, neurological disorders, or prior competition experience in gymnastics.

Before recruitment, we conducted a power analysis using G^*^Power (ANOVA, between-subjects design, α = 0.05, power = 0.80, effect size *f* = 0.25). The analysis indicated a minimum of 58 participants per group. Allowing for an anticipated dropout rate of about 10%, we initially recruited 260 students. Twenty-eight were later excluded because of scheduling conflicts, missing data, or video recording failures, resulting in 232 valid cases.

There were clear theoretical and methodological reasons for focusing on novice university students. First, this group is relatively homogeneous in baseline skill level, which reduces variability due to prior technique, coaching history, or entrenched movement patterns. This increases the sensitivity to feedback manipulations, particularly for motivational and metacognitive processes. Second, research on SDT and skill acquisition suggests that autonomy support and spaced feedback are especially influential in early learning, when learners rely heavily on augmented feedback and are in the initial stages of building motor schemas. Finally, the vaulting task was adjusted in difficulty and is commonly used in university gymnastics courses, ensuring both safety and ecological validity while capturing a learning stage where feedback effects are most pronounced.

All participants provided written informed consent. The study protocol was approved by the university ethics committee (Approval No. 102772024RT201) and complied with the principles of the Declaration of Helsinki.

### 2.2 Experimental task and design

The experimental task was a goat stride vault, a multi-phase gymnastics vaulting skill involving a run-up, take-off, hand support on the apparatus, hip lift with leg separation, and a controlled landing. The task was selected because it requires coordinated execution across several movement phases while remaining feasible and safe for novice university learners after standard instruction and protective setup. In terms of motor-learning demands, it represents a skill of moderate complexity, making it suitable for examining how feedback frequency and feedback control influence performance, retention, and transfer in an ecologically valid instructional setting.

To ensure safety, we adjusted apparatus height, used stepwise demonstrations, and provided thick landing mats. These measures allowed novices to perform the task safely while maintaining a realistic instructional context. Importantly, the multi-phase structure of the skill provides rich opportunities for KP cues while still allowing learners without formal training to complete the task with moderate guidance. This makes it an ideal context for testing the impact of feedback frequency and autonomy on performance and psychological processes.

Movement performance was recorded using four DJI Action 3 high-speed cameras (frame rate ≥ 60 fps). Two cameras were positioned in the sagittal plane on each side of the apparatus, approximately 3 m away and 1.7 m high, to capture key technical elements. A front-view camera recorded landing stability. A fourth camera was positioned at an oblique rear angle to capture whole-body alignment and flight-phase coordination. When available, a timing gate system was used to monitor approach speed for procedural control; these data were not used in the primary analyses. All video recordings were synchronized and time-stamped using the “ai4sports System.”

Video-based KP feedback was presented on a 13-inch laptop. Each KP episode consisted of a 3–5 s slow-motion replay of the immediately preceding attempt (video-based KP), accompanied by two standardized verbal cues selected from a pre-defined feedback manual targeting key technical elements (e.g., trunk alignment at take-off/support; shoulder angle at hand contact; hip lift and leg separation; landing stability; see Supplementary Appendix S1). To ensure consistency, the same trained instructor delivered feedback across all groups using the same cue library and delivery script. No KR (e.g., score feedback) was provided during practice beyond the KP manipulation. Experimental manipulations focused solely on feedback frequency and feedback control, as described below.

We used a 2 × 2 between-subjects design. The independent variables were feedback frequency (high vs. low) and feedback control (instructor-controlled vs. self-controlled). Participants were stratified by gender and baseline performance (pre-practice score) using rank-order blocks, and then randomly assigned within strata to one of the four conditions.

#### 2.2.1 Frequency manipulation

- High-frequency feedback (KP availability): KP was available after each trial (i.e., an opportunity to access KP after every attempt).
- Low-frequency feedback (limited opportunities): KP opportunities were limited to two trials per 10-trial block (≈20%).

Accordingly, in the present study, the frequency manipulation was defined in terms of KP availability/opportunity constraints rather than compulsory KP receipt on every trial.

#### 2.2.2 Control manipulation

- Instructor-controlled condition: KP was delivered according to an a priori pre-set schedule. In the low-frequency instructor-controlled condition, KP was delivered on Trials 5 and 10 within each 10-trial block (20%), applied identically across participants and blocks. The implementation of these schedules is documented via logged process indicators (actual feedback frequency and feedback delay).
- Self-controlled condition: Learners decided on which trials to receive KP using a fixed number of feedback credits to preserve the intended frequency manipulation. In the low-frequency condition, each learner received two credits per 10-trial block (20%), and no additional KP was available once credits were used. In the high-frequency condition, KP was available after every trial, and learners decided whether to access it. Thus, the planned manipulation reflected KP availability, whereas actual access behavior could vary and was logged for transparency. Actual access/request behavior was logged; the resulting actual feedback frequency and self-request proportion by condition are reported in Supplementary Table S4.

A fully yoked (trial-by-trial) design is commonly used to equate both quantity and timing of feedback between self-controlled and externally controlled conditions. In the present 2 × 2 factorial design, our primary aim was to examine autonomy over feedback access under different availability constraints (high availability vs. limited opportunities). Accordingly, the present design should be interpreted as examining feedback control under differing KP-availability constraints rather than as a fully timing-matched autonomy manipulation. We therefore standardized the instructor-controlled timing via an a priori schedule and constrained self-controlled opportunities via a credit system, while additionally reporting logged process indicators (Supplementary Table S4) to document how feedback access unfolded in practice. We acknowledge that a trial-by-trial yoked timing control could further isolate autonomy effects from timing effects; this is noted as a limitation and a direction for future research.

The experiment consisted of three phases: acquisition, retention, and transfer. During acquisition, participants completed 3 blocks of 10 trials (30 trials total) in a single session. The retention test was conducted 48 h later and consisted of 10 trials. Immediately afterward, participants completed a transfer test, also with 10 trials, in which apparatus height was increased by 10 cm for all participants, while the number of approach steps was held constant. Details on warm-up, demonstrations, pacing of trials, and data recording are described in the procedure section.

### 2.3 Procedure

Before acquisition, participants completed a standardized warm-up and received a live demonstration plus key safety instructions. They were instructed to prioritize correct technical execution (run-up rhythm, take-off mechanics, stable support, controlled hip lift/leg separation, and safe landing) rather than maximal speed. One familiarization trial was permitted. Acquisition consisted of 3 blocks of 10 trials (30 trials total) with standardized rest intervals. Retention testing occurred 48 h later (10 trials) without augmented feedback. Transfer testing immediately followed (10 trials) with a standardized task modification (apparatus height + 10 cm for all participants, with approach steps held constant) to evaluate generalization.

#### 2.3.1 Trial recording

For each trial, we recorded: trial ID, block number, experimental group, time stamp, apparatus height, number of approach steps, whether feedback was given (feedback_given, 0/1), feedback type (feedback_type: KP), feedback delay (feedback_delay), whether feedback was self-requested (0/1), and judge score (0–10).

In the self-controlled groups, participants also reported their reasons for requesting feedback. Two independent raters categorized these reasons as: (a) perceived clear error; (b) uncertainty about technique; or (c) desire to confirm a successful performance. Interrater agreement was 94.8% (110/116); Cohen's κ was 0.898 (95% CI 0.821–0.965), indicating excellent agreement beyond chance.

### 2.4 Measures

#### 2.4.1 Gymnastics performance

Two FIG-certified judges independently scored each trial using an adapted rubric specifying deductions/criteria for run-up rhythm, take-off angle, body alignment and extension, hand support quality, hip lift/leg separation, and landing stability (0–10 scale). To minimize expectancy and order effects, all trials were video-recorded and exported as de-identified clips (anonymous IDs), and judges were blind to experimental condition and test phase. Importantly, clips were pooled across participants and phases and presented in a fully randomized order, so judges did not view attempts in chronological sequence and had no information that could reveal the time point (acquisition vs. retention vs. transfer). Interrater reliability was excellent (ICC[2,k] =0.92, 95% CI [0.88,0.95]).

Phase-specific performance indices were computed as:

- **Acquisition:** mean score of the last 5 trials.
- **Retention:** mean score of the 10 retention trials.
- **Transfer:** mean score of the 10 transfer trials.

#### 2.4.2 Manipulation checks and log-based indicators

At the end of each phase, participants completed two items on a 7-point Likert scale as manipulation checks:

1. “Feedback was frequent.”
2. “I could decide when to receive feedback.”

In addition, system logs were used to extract objective process indicators: actual feedback frequency (proportion of trials receiving KP), feedback delay (in milliseconds), and self-request rate (percentage of trials in which feedback was requested). These variables were used in exploratory process analyses. Feedback delay was recorded as a process-quality indicator to verify that between-condition differences were not attributable to systematic timing differences in feedback delivery.

#### 2.4.3 Autonomous motivation

Autonomous motivation was assessed using the second version of the Sport Motivation Scale (SMS-II) (Pelletier et al., 2013). A composite score was computed by combining intrinsic motivation and identified regulation subscales. Internal consistency was high across phases (Cronbach's α =0.87–0.89). For mediation analyses, we primarily used the mean score from the acquisition phase; motivation scores from the retention phase were included in sensitivity analyses.

#### 2.4.4 Metacognitive strategies

Metacognitive strategies were measured using an adapted version of the Metacognitive Awareness Inventory (Schraw and Dennison, 1994), including three subscales: planning, monitoring, and evaluation. Internal consistency was good (planning α =0.79, monitoring α =0.81, evaluation α =0.76). Subscale scores were z-standardized and averaged to form a composite metacognitive strategy index.

#### 2.4.5 Covariates / Screening

Covariates included age, gender, and baseline skill level (pre-practice performance). Approach speed was monitored for procedural control when available but was not included in the primary analyses. Baseline group differences are reported in the Results section; because a small baseline difference was observed, baseline performance was controlled as a covariate in subsequent analyses.

### 2.5 Data analysis

Statistical analyses were conducted using SPSS 25 and Amos 26. The significance level was set at α = 0.05 (two-tailed). Exact *p*-values were reported when *p* ≥ 0.001; values below this threshold were reported as *p* < 0.001. Confidence intervals were also reported to three decimal places. When assumptions underlying statistical tests were violated, appropriate robust or corrected methods were applied.

#### 2.5.1 Preprocessing

The overall rate of missing data was low ( ≤ 1.5% for all variables). For SEM analyses, missing values were handled using full information maximum likelihood (FIML). We also ran complete-case analyses to check robustness. Normality was evaluated using Q–Q plots and skewness and kurtosis statistics (|skew| ≤ 2.0, |kurtosis| ≤ 7.0). For repeated-measures variables, Mauchly's test was used to assess sphericity. When sphericity was violated, Greenhouse–Geisser corrections were applied and the ε value was reported.

#### 2.5.2 Primary inferential models

To examine phase effects and group differences, we conducted a mixed-design ANOVA with baseline performance as a covariate with:

- Between-subjects factors: feedback frequency (high vs. low) and feedback control (instructor-controlled vs. self-controlled);
- Within-subjects factor: phase (acquisition, retention, transfer).

All reported statistics for this analysis were adjusted for baseline performance to minimize potential bias from pre-practice differences. We reported *F* values, degrees of freedom, exact *p*-values, and partial η^2^. When significant interactions emerged, Bonferroni-corrected simple-effects analyses were used to compare relevant subgroup differences across phases.

#### 2.5.3 Correlational analyses

Pearson correlations with Holm correction were used to examine associations among retention and transfer performance, autonomous motivation, metacognitive strategies, actual feedback frequency, feedback delay, and self-request rate. We additionally computed partial correlations (and regression estimates) controlling for condition assignment and baseline performance; results are reported in Supplementary Table S2.

#### 2.5.4 Sequential mediation model (theoretical model)

To test the hypothesized sequential mediation pathway, we constructed SEM models in which feedback conditions (frequency × control) predicted performance outcomes via autonomous motivation (M1) and metacognitive strategy use (M_2_). Separate models were estimated for retention and transfer performance. We used maximum likelihood estimation with robust standard errors. Model fit was evaluated using χ^2^/df, CFI, TLI, RMSEA, and SRMR (Hu and Bentler, 1999; Kline, 2023). Baseline performance, age, gender, and feedback delay were included as covariates. Indirect effects were estimated using 5,000 bias-corrected bootstrap samples, and 95% confidence intervals were reported. To evaluate H5, we conducted an exploratory moderated mediation analysis using multi-group SEM by feedback frequency (high vs. low), and compared bootstrapped indirect effects across frequency groups (see Supplementary material for exploratory models and supporting adjusted-association analyses).

We specified predictors in two ways:

1. **Categorical predictors (X):** effect-coded variables for control (self-controlled = 1, instructor-controlled = −1) and frequency (low-frequency = 1, high-frequency = −1). Interaction terms (frequency × control) were explored in auxiliary models and are reported in the Supplement when relevant.
2. **Continuous predictors (X; robustness check):** actual feedback frequency and self-request rate (log-transformed) were used instead of categorical variables to test the robustness of the findings.

Covariates included baseline performance, age, gender, and feedback delay. We reported unstandardized regression coefficients (b), standard errors (SE), bias-corrected 95% CIs, direct effects, and model *R*^2^ values.

#### 2.5.6 Sensitivity and supplementary analyses

To further evaluate the robustness of the proposed mechanism and address potential model specification concerns, we conducted the following supplementary analyses (details reported in the Supplementary material):

- Reverse-order mediation (sequence robustness): We estimated an alternative model reversing the mediator order (M_2_ → M1) to evaluate whether the assumed sequential pathway (M1 → M_2_) was uniquely supported.
- Parallel mediation (robustness check): We also estimated an exploratory parallel mediation model, in which autonomous motivation and metacognitive strategy use were specified as concurrent mediators, to examine whether a non-sequential specification provided a better overall account of the data.
- Exploratory moderated mediation for H5 (frequency-dependent indirect effects): We examined whether indirect effects differed across feedback-frequency conditions using multi-group SEM (high vs. low frequency) and compared bootstrapped indirect effects across groups (i.e., exploratory moderated mediation).
- Model-robustness checks: We conducted additional bootstrap-based robustness checks (percentile-based confidence intervals with 10,000 resamples) and complete-case analyses to verify that conclusions were not sensitive to missing-data handling or CI estimation choices.

Baseline performance was included as a covariate in all mediation analyses to minimize potential bias from pre-practice differences (baseline characteristics are reported in Supplementary Table S1).

## 3 Results

### 3.1 Measurement model

Because the mediation model relies on valid measurement of the latent constructs, we first evaluated the measurement model using CFA before testing the hypothesized mediation pathways. A two-factor model specifying autonomous motivation and metacognitive strategies as separate latent constructs showed an overall acceptable fit, χ^2^(89) = 280.09, χ^2^/df = 3.15, CFI = 0.957, TLI = 0.949, RMSEA = 0.096. All standardized factor loadings were substantial (0.773–0.949). Composite reliability (CR) was excellent for both constructs (motivation CR = 0.969; metacognition CR = 0.959), and average variance extracted (AVE) values exceeded the 0.50 criterion (motivation AVE = 0.836; metacognition AVE = 0.723), indicating strong convergent validity.

To examine discriminant validity, we compared the two-factor model with a more parsimonious one-factor model in which all items loaded on a single latent construct. The one-factor model showed significantly poorer fit, χ^2^(90) = 321.90, CFI = 0.947, TLI = 0.939, RMSEA = 0.106, and a chi-square difference test confirmed that the two-factor model fit the data significantly better, Δχ^2^(1) = 41.81, *p* < 0.001. However, the latent correlation between autonomous motivation and metacognitive strategies was very high (*r*≈0.96), suggesting substantial overlap between the two constructs in this sample. Because both mediators were assessed using same-session self-reports, common-method inflation cannot be fully ruled out. Therefore, although the two constructs were empirically distinguishable, the sequential pathway should be interpreted with caution and as theory-consistent rather than as definitive evidence of causal ordering. To avoid over-claiming, we additionally report reverse-order and sensitivity models in the Supplement.

Regarding baseline equivalence, a 2 (frequency: high vs. low) × 2 (control: instructor-controlled vs. self-controlled) ANOVA on pre-practice performance showed no main effect of frequency, *F*_(1, 228)_ = 2.30, *p* = 0.131, and no frequency × control interaction, *F*_(1, 228)_ = 1.52, *p* = 0.219. However, the main effect of control was small but significant, *F*_(1, 228)_ = 3.91, *p* = 0.049, partial η^2^ =0.017, indicating that pre-practice performance in the self-controlled condition was slightly higher. This small difference was statistically controlled in all subsequent primary analyses (including the mixed ANOVA and SEM models), and given its negligible effect size, it is unlikely to affect the interpretation of the main findings. Group-specific baseline means (M ± SD) are reported in Supplementary Table S1.

### 3.2 Manipulation check ratings

To verify the effectiveness of the experimental manipulations, we compared manipulation-check ratings across conditions.

Feedback frequency. Participants in the high-frequency condition reported significantly higher perceived feedback frequency than those in the low-frequency condition, *t*_(189.53)_ = −25.44, *p* < 0.001.

Feedback control. Participants in the self-controlled condition reported significantly greater perceived autonomy over feedback timing than those in the instructor-controlled condition, *t*_(223.76)_ = 33.06, *p* < 0.001.

Together, these results indicate that both manipulations operated as intended.

Request reasons (self-controlled conditions). Participants' reasons for requesting KP were coded as (a) perceived clear technique error, (b) uncertainty about execution, or (c) confirmation of a successful attempt. Across all self-requested KP events pooled across the two self-controlled groups (*N* = 116), the distribution was: perceived error = 20.7% (*n* = 24), uncertainty = 62.1% (*n* = 72), and confirm success = 17.2% (*n* = 20). Interrater agreement was 94.8% (110/116); Cohen's κ was 0.898 (95% CI 0.821–0.965), indicating excellent agreement beyond chance. Detailed counts are provided in Supplementary Table S3.

### 3.3 Mixed-design ANOVA

**Figure 2:**
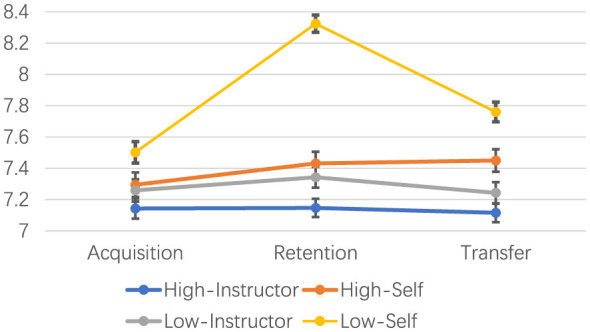
Mean performance trajectories across acquisition, retention, and transfer phases for the four feedback conditions. Error bars represent standard errors.

Overall descriptive statistics are presented in Table 1; group-specific means ± SD by phase are reported in Table 2 and visualized in Figure 2. Because the performance rubric uses a restricted 0–10 scale and the sample consisted of beginners performing a standardized task, between-participant variability was modest (SD ≈ 0.5); therefore, we emphasize covariate-adjusted comparisons and effect sizes (e.g., partial η^2^) rather than interpreting SD magnitude alone. Baseline performance was included as a covariate to account for the small pre-practice difference between control conditions. To examine the effects of feedback frequency, feedback control, and learning phase, we conducted a 2 (frequency: high vs. low) × 2 (control: instructor-controlled vs. self-controlled) × 3 (phase: acquisition, retention, transfer) mixed-design ANOVA with baseline performance included as a covariate (to account for the small pre-practice difference between control conditions), with phase as the within-subjects factor.

**Table 1:** Descriptive statistics of psychological variables.

Variable	M	SD
Autonomous motivation	4.98	1.03
Metacognitive strategies	3.00	0.83

*M*, mean; *SD*, standard deviation.

**Table 2:** Means ± SD of performance scores by condition and phase.

Condition	*n*	Acquisition (M ± SD)	Retention (M ± SD)	Transfer (M ± SD)
High-frequency/Instructor-controlled	58	7.143 ± 0.492	7.147 ± 0.447	7.116 ± 0.461
High-frequency/Self-controlled	58	7.295 ± 0.597	7.431 ± 0.569	7.450 ± 0.547
Low-frequency/Instructor-controlled	58	7.259 ± 0.539	7.343 ± 0.505	7.243 ± 0.524
Low-frequency/Self-controlled	58	7.502 ± 0.521	8.324 ± 0.420	7.760 ± 0.479

“High-frequency” denotes high KP availability (opportunity after each trial), whereas “low-frequency” denotes limited opportunities (two trials per 10-trial block). Values are means ± SD.

Mauchly's test indicated that the sphericity assumption for the within-subjects factor (phase) was violated, W = 0.963, χ^2^(2) = 8.556, *p* = 0.014. Therefore, Greenhouse-Geisser corrections with ε =0.964 were applied to all statistical effects involving the phase factor.

The analysis revealed a significant main effect of phase, *F*_(1.929, 439.735)_ = 46.660, *p* < 0.001, partial η^2^ = 0.170. There was a significant phase × frequency interaction, *F*_(1.929, 439.735)_ = 25.635, *p* < 0.001, partial η^2^ = 0.101, and a significant phase × control interaction, *F*_(1.929, 439.735)_ = 56.324, *p* < 0.001, partial η^2^ = 0.198. The three-way interaction among phase, frequency, and control was also significant, *F*_(1.929, 439.735)_ = 20.995, *p* < 0.001, partial η^2^ = 0.084.

For between-subjects effects, the main effect of frequency was significant, *F*_(1, 228)_ = 10.508, *p* < 0.001, partial η^2^ = 0.044, as was the main effect of control, *F*_(1, 228)_ = 11.052, *p* < 0.001, partial η^2^ = 0.046. The interaction between frequency and control was not significant, *F*_(1, 228)_ = 1.594, *p* = 0.208, partial η^2^ = 0.007. All reported partial η^2^ values reflect the magnitude of adjusted effect sizes for the respective factors.

### 3.4 Correlational analyses

Table 3 reports Pearson correlations among psychological variables, performance outcomes, and feedback behavior indices. Autonomous motivation and metacognitive strategies were highly correlated, *r* = 0.965, *p* < 0.001. Both were strongly correlated with the proportion of self-requested feedback, with autonomous motivation-self-requested feedback proportion *r* = 0.925 and metacognitive strategies-self-requested feedback proportion *r* = 0.886 (both *p* < 0.001).

**Table 3:** Pearson correlations among motivation, metacognition, performance, and feedback-related variables.

Variable	1	2	3	4	5	6	7	8
1. Autonomous motivation	—							
2. Metacognitive strategies	0.965^**^	—						
3. Acquisition mean score	0.425^**^	0.430^**^	—					
4. Retention mean score	0.717^**^	0.787^**^	0.829^**^	—				
5. Transfer mean score	0.612^**^	0.619^**^	0.933^**^	0.907^**^	—			
6. Actual feedback frequency	−0.276^**^	−0.428^**^	−0.454^**^	−0.619^**^	−0.479^**^	—		
7. Self-requested feedback proportion	0.925^**^	0.886^**^	0.332^**^	0.615^**^	0.511^**^	−0.192^**^	—	
8. Feedback delay	−0.005	−0.007	−0.038	−0.044	−0.060	0.034	0.021	—

Values are Pearson correlations (two-tailed). ^**^p < 0.01. *N* = 232.

Regarding performance, both autonomous motivation and metacognitive strategies were positively correlated with acquisition, retention, and transfer scores (*r* = 0.425–0.787, all *p* < 0.001), with particularly strong associations with retention and transfer.

Unadjusted correlations among behavioral feedback indices, psychological mediators, and performance are reported in Table 3 and should be interpreted descriptively. Notably, “feedback frequency” in the experimental design reflects a manipulated schedule (limited opportunities vs. high availability), whereas “actual feedback frequency” reflects behavioral variability in the feedback received across practice. Because this behavioral index is partly constrained by the assigned condition, its bivariate association with retention/transfer does not constitute causal evidence and should not be used to reinterpret the manipulated frequency effect. For clarity, we also remind readers that SEM frequency was effect-coded (low = 1, high = −1), such that positive coefficients indicate an advantage for the low-frequency condition. In addition, to separate within-design associations from between-condition differences, we report partial correlations/regression estimates that control for condition assignment (and baseline performance) in the (Supplementary Table S2).

### 3.5 SEM results

#### 3.5.1 Model fit

**Figure 3:**
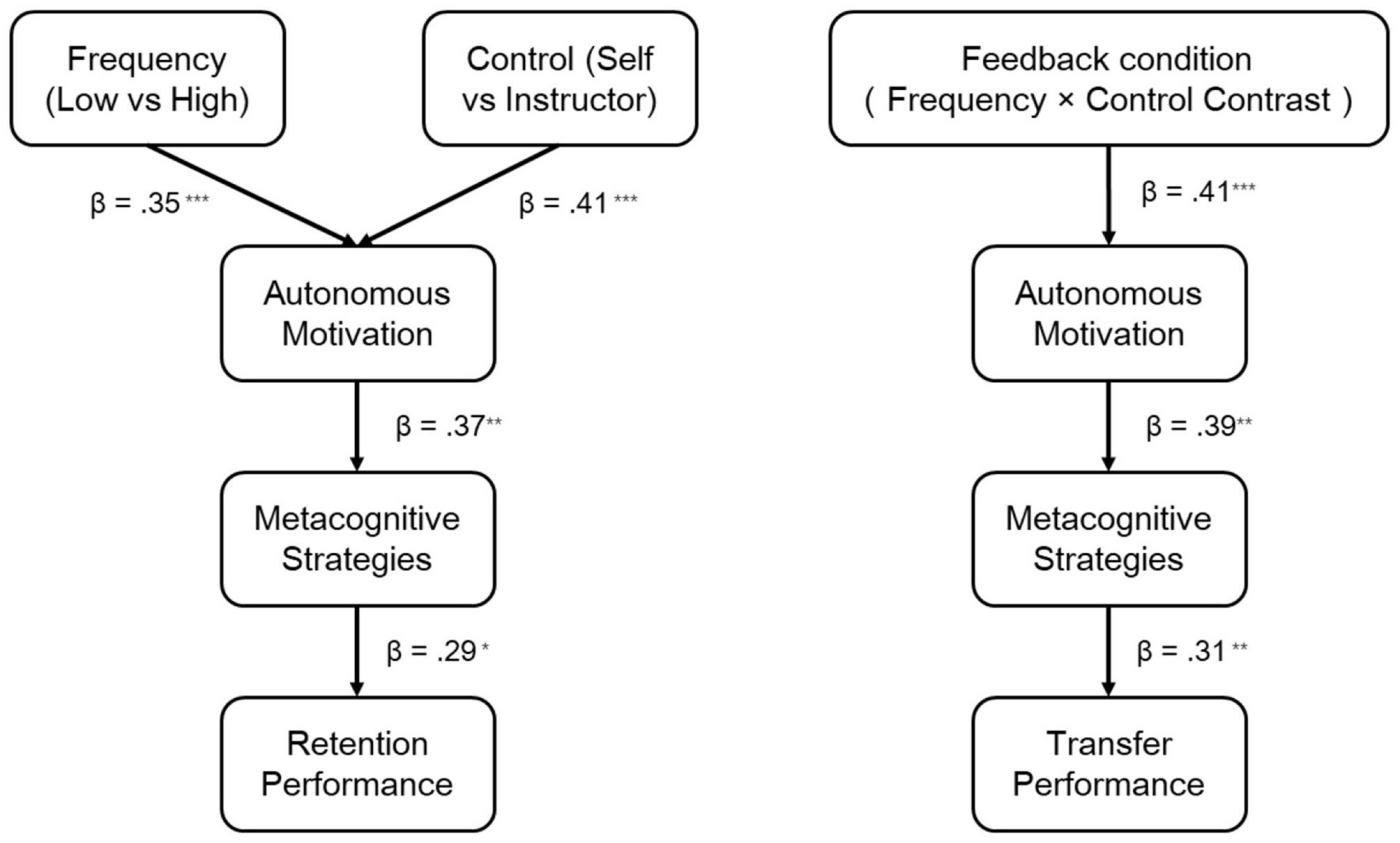
Structural equation models for retention and transfer performance. **(A)** Model testing the hypothesized sequential mediation pathway in the retention phase. **(B)** Model testing the same sequential mediation pathway in the transfer phase. Standardized path coefficients (β) are shown. Solid lines represent significant paths; non-significant direct paths are omitted for clarity. All models controlled for baseline performance, age, gender, and feedback delay. Unstandardized estimates are reported in the text. Standardized coefficients shown. ***p < 0.001, **p < 0.01, *p < 0.05. Non-significant direct paths omitted for clarity.

To further test the hypothesized sequential mediation pathway, we specified two SEMs. Baseline performance was statistically controlled by specifying paths from baseline to all post-practice outcomes in the mediation model. In both models, two effect-coded experimental predictors were entered: feedback control (self-controlled = 1, instructor-controlled = −1) and feedback frequency (low-frequency = 1, high-frequency = −1). Autonomous motivation and metacognitive strategy use were modeled as sequential mediators, and either retention or transfer performance served as the outcome. Baseline performance, age, gender, and feedback delay were included as covariates. We report unstandardized estimates (b), standard errors (SE), and *p* values in the text; bootstrap confidence intervals are provided for indirect effects. For clarity, unstandardized path coefficients (b) with standard errors are reported in the text, whereas standardized coefficients (β) are presented in Figure 3.

The two structural equation models demonstrated acceptable to good fit to the data. For the retention model, the RMSEA value was slightly above the conventional 0.08 cutoff, while the transfer model's RMSEA was well below this threshold; in both models, CFI, TLI, and SRMR indicated good fit, and RMSEA confidence intervals remained within an acceptable range for complex SEM models. These values indicate an overall satisfactory model fit (Table 4).

**Table 4:** Model fit indices for the structural equation models.

Model	χ^2^ (df)	CFI	TLI	RMSEA [90% CI]	SRMR
Final SEM (Retention)	239.49 (90)	0.973	0.969	0.085 [0.072,0.098]	0.023
Final SEM (Transfer)	109.58 (74)	0.958	0.939	0.045 [0.023,0.063]	0.042

#### 3.5.2 Retention model

In the retention model, feedback frequency significantly predicted autonomous motivation (*b* = 0.274, SE = 0.024, *p* < 0.001), and feedback control also significantly predicted autonomous motivation (*b* = 1.290, SE = 0.024, *p* < 0.001). Autonomous motivation significantly predicted metacognitive strategy use (*b* = 0.756, SE = 0.013, *p* < 0.001), and metacognitive strategy use was significantly related to retention performance (*b* = 0.749, SE = 0.038, *p* < 0.001). Autonomous motivation also showed a significant direct association with retention performance when controlling for metacognitive strategy use and covariates (*b* = −0.325, SE = 0.030, *p* < 0.001); this negative coefficient reflects a statistical suppression effect due to the high latent correlation (*r*≈0.96) between autonomous motivation and metacognitive strategy use, rather than a meaningful negative psychological effect. Among the covariates, baseline performance was positively related to retention performance (*b* = 0.637, SE = 0.018, *p* < 0.001), whereas age (*p* = 0.257), gender (*p* = 0.394), and feedback delay (*p* = 0.365) were not significant.

Bias-corrected bootstrap analyses with 5,000 resamples indicated that the total indirect effect of feedback control on retention performance was significant (*b* = 0.312, 95% BC CI [0.278, 0.348], *p* < 0.001), and the total indirect effect of feedback frequency on retention performance was also significant (*b* = 0.066, 95% BC CI [0.054, 0.081], *p* < 0.001). In addition, the indirect effect of autonomous motivation on retention performance via metacognitive strategy use was significant (*b* = 0.567, 95% BC CI [0.505, 0.626], *p* < 0.001).

Because the model included a direct path from autonomous motivation to retention performance, the reported total indirect effects for feedback control and feedback frequency reflect the combined indirect pathways via autonomous motivation alone and via autonomous motivation → metacognitive strategy use.

#### 3.5.3 Transfer model

In the transfer model, feedback frequency significantly predicted autonomous motivation (*b* = 0.274, SE = 0.024, *p* < 0.001), and feedback control also significantly predicted autonomous motivation (*b* = 1.290, SE = 0.024, *p* < 0.001). Autonomous motivation significantly predicted metacognitive strategy use (*b* = 0.756, SE = 0.013, *p* < 0.001). Both metacognitive strategy use (*b* = 0.090, SE = 0.037, *p* = 0.016) and autonomous motivation (*b* = 0.061, SE = 0.029, *p* = 0.037) were positively associated with transfer performance. Among the covariates, baseline performance (*b* = 0.711, SE = 0.018, *p* < 0.001) and gender (*b* = 0.058, SE = 0.021, *p* = 0.006) were positively associated with transfer performance, age was negatively associated with transfer performance (*b* = −0.054, SE = 0.022, *p* = 0.012), whereas feedback delay was not significant (*p* = 0.138).

Bias-corrected bootstrap analyses with 5,000 resamples indicated that the total indirect effect of feedback control on transfer performance was significant (*b* = 0.167, 95% BC CI [0.145, 0.191], *p* < 0.001), and the total indirect effect of feedback frequency on transfer performance was also significant (*b* = 0.036, 95% BC CI [0.028, 0.044], *p* < 0.001). In addition, the indirect effect of autonomous motivation on transfer performance via metacognitive strategy use was significant (*b* = 0.068, 95% BC CI [0.007, 0.129], *p* = 0.029).

#### 3.5.4 Summary of mediation pattern

Across both the retention and transfer models, feedback control and feedback frequency were significantly associated with autonomous motivation, and autonomous motivation was significantly associated with metacognitive strategy use. Bias-corrected bootstrap confidence intervals supported significant total indirect effects of feedback control and feedback frequency on performance outcomes. Because the models included a direct path from autonomous motivation to performance, these total indirect effects represent the combined indirect pathways via autonomous motivation alone and via autonomous motivation → metacognitive strategy use. In addition, as an exploratory robustness check, we also estimated parallel mediation models for retention and transfer. These models showed substantially poorer overall fit than the theory-driven sequential models (Retention parallel: χ^2^ = 412.50, CFI = 0.839, TLI = 0.676, RMSEA = 0.108, SRMR = 0.111; Transfer parallel: χ^2^ = 637.14, CFI = 0.734, TLI = 0.596, RMSEA = 0.175, SRMR = 0.110) and therefore were not retained as the primary analytic specification.

## 4 Discussion

### 4.1 Key findings and theoretical integration

Across multiple sources of evidence, the present study indicates that self-controlled KP feedback and reduced feedback frequency each contributed to more durable learning of a gymnastics skill, consistent with an additive (main-effect) pattern (Chiviacowsky and Wulf, 2002; Winstein and Schmidt, 1990). Descriptively, retention and transfer scores were highest in the self-controlled/low-frequency subgroup, and this direction of effects was consistent with robustness checks (Guadagnoli and Lee, 2004). However, the mixed ANOVA (adjusted for baseline performance) did not support a frequency × control interaction, and our SEMs did not model a latent interaction term. Therefore, we interpret subgroup differences as cumulative benefits of autonomy support and spaced feedback, rather than evidence for a unique “optimal” combination driven by synergistic effects. Consistent with the ANOVA, we found no evidence for a multiplicative interaction in performance outcomes. In addition, feedback control and frequency showed no statistically significant direct effects on retention or transfer; instead, their associations with performance were primarily explained by indirect pathways via autonomous motivation and metacognitive strategy use.

Reduced feedback frequency may have supported learning by attenuating immediate performance dependence and encouraging learners to engage in internal error-detection and self-evaluation processes between feedback episodes (Winstein and Schmidt, 1990; Guadagnoli and Lee, 2004). From an information-processing perspective, spacing KP may encourage learners to maintain and update an internal reference of correct movement, which can strengthen retention and transfer even when acquisition performance is not maximized. In parallel, self-controlled feedback likely enhanced autonomy and task engagement by allowing learners to request KP when it was perceived as most informative or motivationally meaningful (Chiviacowsky and Wulf, 2002). This autonomy-supportive structure may increase perceived competence and ownership of learning, thereby promoting greater persistence and deeper strategic regulation during practice. Together, these interpretations are consistent with H2–H4, which posit that frequency and control primarily shape learning through motivational and metacognitive processes rather than through large direct effects on performance.

The absence of a statistically significant frequency × control interaction in the ANOVA indicates that the effects of feedback frequency and feedback control on performance were not multiplicative in the present dataset. One possibility is that feedback frequency and feedback control may influence learning through partially overlapping mechanisms, and any potential conditional dependency may be too small to detect at the behavioral level in the present design. We emphasize that such conditional interpretations are speculative, and future work using explicitly yoked timing controls and interaction-focused SEM (e.g., latent interaction terms) is needed to test moderation more directly. Another possibility is that between-subject variability and modest effect sizes reduced power to detect an interaction at the behavioral level, even though the direction of effects was consistent across phases. Importantly, the SEM results provide convergent evidence that frequency and control remain distinguishable instructional levers: frequency primarily shaped information-processing demands, whereas control primarily shaped motivational readiness, with both converging on metacognitive strategy use. Accordingly, we refer to the self-controlled/low-frequency condition only as the descriptively highest mean subgroup, and we interpret the overall pattern as independent and cumulative contributions of autonomy support and reduced feedback frequency that operate through motivation- and metacognition-related pathways, rather than as evidence for a “best-performing combination” produced by a true interaction.

More importantly, the SEM results supported the proposed sequential mediation mechanism. Feedback structure influenced performance by first enhancing autonomous motivation, which in turn increased the use of metacognitive strategies, ultimately contributing to both retention and transfer (Efklides, 2011). This mechanism reflects the central role of autonomy posited in Self-Determination Theory (SDT) and the importance of strategy engagement emphasized in metacognitive control models. Although autonomous motivation and metacognitive strategies were strongly correlated in the present study, this pattern is theoretically consistent with current models of self-regulated learning. Given this high overlap, we acknowledge potential multicollinearity and suppression effects in the sequential model; therefore, any attenuated or sign-reversed direct paths are interpreted with caution, and emphasis is placed on the indirect-effect pattern and robustness checks. Motivation provides the energy and direction for engagement, whereas metacognition reflects learners' strategic monitoring and control of their actions. When learners are autonomously motivated, they are more likely to engage in reflective planning, monitoring, and evaluation, leading to a naturally tight functional coupling between the two processes. Importantly, however, the confirmatory factor analysis demonstrated that a two-factor measurement model fit the data significantly better than a one-factor alternative, and both constructs showed excellent convergent validity. These results indicate that the high association reflects meaningful psychological interdependence rather than measurement redundancy, supporting the conceptual distinction between motivation and metacognition. The findings also show that feedback frequency and feedback control function as complementary instructional levers: frequency shapes information-processing demands, whereas control shapes motivational readiness. By integrating behavioral logs, psychological measures, and performance outcomes, this study provides multi-level evidence for a motivation-cognition-performance chain in motor learning. At the same time, the observed effects were generally small-to-moderate, and SEM results are constrained by model fit and the cross-sectional measurement of self-report mediators. Therefore, we interpret the indirect pathways as theory-consistent and hypothesis-aligned rather than as definitive evidence of causal ordering, and we emphasize effect sizes and robustness checks over dichotomous significance alone.

### 4.2 Mechanistic interpretation, sequential mediation, and theoretical implications

Here, “mechanistic interpretation” refers to our hypotheses-driven mediation account tested in the SEM: feedback structure (frequency and control) influences retention and transfer primarily through indirect pathways, first via autonomous motivation and then via metacognitive strategy use. This sequential pathway operationalizes the proposed motivation-cognition-performance mechanism in motor learning. The mediation results illustrate how motivational and cognitive mechanisms jointly support skill learning. Self-controlled feedback satisfies learners' need for autonomy and enhances self-determined motivation (Wulf and Lewthwaite, 2016). Higher motivation then encourages greater engagement in planning, monitoring, and evaluation—key metacognitive strategies that enable skill refinement and error correction (Efklides, 2011).

Although a frequency × control interaction was theoretically expected, it did not emerge in the ANOVA. With respect to H5, we conducted an exploratory moderated mediation analysis using multi-group SEM by feedback frequency (high availability vs. limited opportunities) and compared bootstrapped indirect effects across groups. This exploratory analysis did not provide clear evidence that the sequential indirect pathway was stronger under low-frequency feedback; accordingly, H5 was not supported and is not emphasized as a central conclusion. Limited sensitivity to higher-order differences, sample homogeneity, and the substantial overlap between the mediators may have further constrained the detection of frequency-contingent indirect effects. Nevertheless, across both frequency conditions, SEM consistently supported the hypothesized sequential indirect pathway (autonomous motivation → metacognitive strategy use → performance), aligning with H4.

Within this sequential model, autonomous motivation showed a negative direct association with retention performance when metacognitive strategy use was taken into account. This pattern should not be interpreted as evidence of a detrimental role of autonomous motivation in learning, but rather as a statistical suppression effect driven by severe multicollinearity between autonomous motivation and metacognitive strategy use (latent correlation *r*≈0.96). In this multivariate model, the substantial shared predictive variance between the two constructs is primarily absorbed by metacognitive strategy use, leading to an artificially negative direct coefficient for autonomous motivation—a mathematical artifact rather than a meaningful psychological phenomenon. Notably, the zero-order bivariate correlation between autonomous motivation and retention performance remains significantly positive (Table 3), confirming that autonomous motivation's predictive value for retention is largely subsumed by metacognitive strategy use in the full sequential mediation model. This statistical artifact does not undermine the robustness of the hypothesized sequential mediation pathway, as the indirect effect of autonomous motivation on retention performance via metacognitive strategy use remains strongly significant and positive. Overall, the pattern of estimates was consistent with additive advantages of greater learner control and reduced feedback availability, without evidence for a multiplicative interaction between the two factors. Thus, the absence of a statistical interaction does not undermine the theoretical model (Kline, 2023).

With regard to the hypothesized mediation pathways, neither the single mediation via autonomous motivation (H2) nor via metacognitive strategy use (H3) was significant, whereas the sequential mediation pathway (motivation → metacognition; H4) received robust support. This means that autonomous motivation and metacognitive strategies cannot independently explain the effect of feedback structure (frequency and control); only when they operate in the hypothesized sequential order do they fully link feedback conditions to learning performance (Katsantonis, 2020). This pattern supports a layered view of learning mechanisms: motivation functions as the initiator of self-regulated learning, while metacognitive strategies provide the cognitive pathway through which learning occurs.

The findings also extend evidence related to the guidance hypothesis. Low-frequency feedback gives learners more opportunities to use intrinsic cues for error detection and self-correction, contributing to more generalized motor schemas. This mechanism aligns with the challenge point framework's emphasis on optimizing informational load (Guadagnoli and Lee, 2004).

Overall, the study demonstrates an integrated “motivated metacognition” pathway. SEM results strengthen the applicability of this framework in skill-learning contexts (Kline, 2023) and highlight the central role of psychological processes in motor learning. The findings contribute new empirical support for integrating SDT, metacognitive theory, and motor learning theory.

### 4.3 Practical implications for teaching and coaching

The findings offer clear practical guidance for teaching gymnastics and other complex motor skills. First, our results reaffirm the long-standing guidance-hypothesis view that more feedback is not necessarily better for durable learning (Salmoni et al., 1984; Winstein and Schmidt, 1990), while extending this principle by highlighting a complementary motivational–metacognitive pathway. Although high-frequency KP can improve immediate performance during acquisition, it appears to hinder retention and transfer, consistent with research on over-guidance (McKay et al., 2022b).

Second, self-controlled feedback allows learners to determine when feedback is most useful, increasing the efficiency of feedback use and fostering active engagement (Bacelar et al., 2022). Learners typically request feedback when uncertain or when they detect an error, making feedback more targeted and informative.

In practice, coaches may provide video-based KP feedback on a low-frequency schedule while allowing learners some autonomy in choosing the timing (Potdevin et al., 2018). Coaches can also prompt metacognitive reflection through guiding questions such as “What will you focus on next time?” or “When do you think you need feedback?” Such strategies enhance technical learning while supporting autonomy and self-reflection—capacities closely linked to long-term physical literacy (Mödinger et al., 2022).

### 4.4 Limitations and directions for future research

Several limitations should be noted. First, the padded box-horse task is multi-phase and moderately complex for novices, yet it remains simplified relative to full competitive vault routines; therefore, replication across a broader range of gymnastics skills is warranted. Moreover, because the sample comprised university beginners, the findings may not generalize to children, elite athletes, or more advanced apparatus skills. Future research should test broader populations and skill types. In addition, the acquisition phase included only 30 trials, which likely reflects early-stage learning rather than late-stage or asymptotic learning. Future work should extend practice duration and examine whether the motivational-metacognitive pathway persists across longer training periods.

Second, autonomous motivation and metacognitive strategies were highly correlated (Katsantonis, 2020), suggesting potential conceptual overlap despite the SEM evidence for their statistical separability. This close association may simply reflect their coordinated functioning in early motor learning. Nevertheless, future studies should use multi-method approaches to more clearly disentangle their unique contributions. Although a small baseline difference was observed between control conditions, the effect size was minimal and baseline performance was statistically controlled, reducing the likelihood that it influenced the main findings.

Third, motivational and metacognitive measures were assessed at the phase level and did not capture trial-by-trial dynamics. Future studies could use multilevel SEM (MSEM), eye-tracking, or physiological data to describe fluctuations in motivation and cognition during practice.

Fourth, despite standardized feedback scripts and KP cues, small variations in coach delivery or video selection may introduce ecological noise (Potdevin et al., 2018). Moreover, the study could not fully rule out potential bidirectional relations between motivation and metacognition (MacKinnon and Luecken, 2008). Longitudinal cross-lagged designs may help clarify causal ordering.

Finally, because mediation effects are sensitive to sample size, the study may have limited power to detect more complex indirect or moderated effects (McNeish, 2016). Larger samples, multi-site collaborations, or Bayesian SEM could improve estimate stability in future research.

Despite these limitations, the study provides systematic evidence showing how autonomy support and spaced feedback promote skill learning through motivational and metacognitive mechanisms. The findings offer valuable insights for designing instruction for complex motor skills.

## 5 Conclusion

This study shows that self-controlled KP feedback and reduced feedback frequency are each associated with more durable learning outcomes, consistent with additive rather than synergistic effects. This advantage appeared in both retention and transfer performance. The results further support a plausible, theory-consistent motivational-metacognitive account, in which autonomy support and spaced KP are linked to learning outcomes through autonomous motivation and metacognitive strategy use within the limits of the present design.

Practically, the findings provide actionable guidance for teaching gymnastics and other complex skills. Coaches may grant learners some autonomy over feedback timing and provide concise, video-based KP cues at appropriate intervals, while ensuring safety and core technical accuracy. These findings may inform feedback design in educational and sport-training settings by supporting autonomous learning and coaching efficiency in skill acquisition.

## Data Availability

The datasets presented in this study can be found in online repositories. The names of the repository/repositories and accession number(s) can be found at: https://osf.io/c68kb/overview?view_only=827258af038d4547ae2339a5acdd6a11.
