# Supplementary material for: Self-controlled and low-frequency KP feedback enhances gymnastics skill learning via motivation and metacognition
Source: Front Psychol. 2026 Apr 23;17:1769157. doi: 10.3389/fpsyg.2026.1769157 (PMC13149236; doi:10.3389/fpsyg.2026.1769157)
Supplement: Supplementary file 1 [file Table_1.docx]

Supplementary：

**Table S1. Baseline characteristics by feedback condition**

| **Characteristic** | **High-frequency / Instructor-controlled (n=58)** | **High-frequency / Self-controlled (n=58)** | **Low-frequency / Instructor-controlled (n=58)** | **Low-frequency / Self-controlled (n=58)** |
| --- | --- | --- | --- | --- |
| Age (years), mean ± SD | 18.98 ± 0.48 | 18.92 ± 0.48 | 18.84 ± 0.50 | 18.80 ± 0.51 |
| Sex = 1, n (%) | 29 (50.0%) | 29 (50.0%) | 29 (50.0%) | 29 (50.0%) |
| Sex = 0, n (%) | 29 (50.0%) | 29 (50.0%) | 29 (50.0%) | 29 (50.0%) |
| Baseline performance, mean ± SD | 7.15 ± 0.52 | 7.21 ± 0.70 | 7.18 ± 0.59 | 7.43 ± 0.59 |

**Note.** Sex was coded as 1/0 in the dataset (see raw data). Baseline performance refers to the pre-practice score.

**Table S2. Partial correlations between key variables after controlling for condition assignment and baseline performance (and sensitivity adjustment for age and sex)**

Panel A. Controlling for condition assignment + baseline performance

| **Predictor variable** | **Outcome variable** | **Partial r** |
| --- | --- | --- |
| Autonomous motivation | Metacognitive strategy use | 0.632 |
| Autonomous motivation | Retention performance | 0.495 |
| Autonomous motivation | Transfer performance | 0.361 |
| Metacognitive strategy use | Retention performance | 0.543 |
| Metacognitive strategy use | Transfer performance | 0.285 |
| Actual feedback frequency | Retention performance | −0.170 |
| Actual feedback frequency | Transfer performance | −0.200 |
| Self-request proportion | Retention performance | ≈0.00 |
| Self-request proportion | Transfer performance | ≈0.00 |

Panel B. Controlling for condition assignment + baseline performance + age + sex

| **Predictor variable** | **Outcome variable** | **Partial r** |
| --- | --- | --- |
| Autonomous motivation | Metacognitive strategy use | 0.63 |
| Autonomous motivation | Retention performance | 0.50 |
| Autonomous motivation | Transfer performance | 0.36 |
| Metacognitive strategy use | Retention performance | 0.54 |
| Metacognitive strategy use | Transfer performance | 0.29 |
| Actual feedback frequency | Retention performance | −0.17 |
| Actual feedback frequency | Transfer performance | −0.20 |
| Self-request proportion | Retention performance | ≈0.00 |
| Self-request proportion | Transfer performance | ≈0.00 |

**Note.** Values are partial Pearson correlations (r). Panel A controls for experimental condition assignment (frequency and control) and baseline performance. Panel B additionally controls for age and sex (0/1 coding as in the dataset). “Actual feedback frequency” denotes the proportion of acquisition trials receiving KP; “self-request proportion” denotes the proportion of acquisition trials in which feedback was requested. Retention and transfer denote post-acquisition performance scores.

**Table S3. Distribution of self-request reasons in self-controlled conditions**

| **Request reason** | **n** | **%** |
| --- | --- | --- |
| Perceived clear error | 24 | 20.7 |
| Uncertainty about technique | 72 | 62.1 |
| Confirmation of success | 20 | 17.2 |
| **Total** | **116** | **100.0** |

**Note.** Reasons were coded for all self-requested KP events in self-controlled conditions. Two independent raters coded each event; raw agreement was 94.8% (110/116) and Cohen’s κ = 0.898 (95% CI 0.821–0.965).

Table S4. Manipulation and process indicators by feedback condition (mean ± SD)

| **Indicator (mean ± SD)** | **High-frequency / Instructor-controlled (n=58)** | **High-frequency / Self-controlled (n=58)** | **Low-frequency / Instructor-controlled (n=58)** | **Low-frequency / Self-controlled (n=58)** |
| --- | --- | --- | --- | --- |
| Actual feedback frequency | 0.569 ± 0.119 | 0.586 ± 0.093 | 0.298 ± 0.064 | 0.269 ± 0.063 |
| Self-request proportion | 0.247 ± 0.078 | 0.602 ± 0.101 | 0.253 ± 0.090 | 0.697 ± 0.071 |
| Feedback delay | 51.980 ± 21.253 | 50.430 ± 19.808 | 49.140 ± 18.164 | 51.720 ± 17.586 |

**Note.** Actual feedback frequency denotes the proportion of acquisition trials on which KP was actually presented (delivered and/or accessed/viewed), as recorded by the system log. Self-request proportion denotes the proportion of acquisition trials on which learners attempted to request KP (e.g., button presses), regardless of whether KP was delivered under the condition’s schedule. Feedback delay reflects the latency between trial completion and KP presentation (units as recorded in the system log). Values are means ± SD.

**Appendix S1. Pre-defined KP feedback manual (cue library and delivery script)**

How it was used in the study. Each KP episode consisted of a 3–5 s slow-motion replay of the immediately preceding attempt plus two standardized cues selected from the library below, targeting the most salient errors observed on that trial. Cues were delivered in a neutral, instructional tone and focused on movement execution (KP) rather than outcome (no KR).

Table S1A. Cue library

| **Technical element (KP target)** | **Typical observable error pattern in video** | **Standardized cue option A (short)** | **Standardized cue option B (short)** | **What to do next (action focus)** |
| --- | --- | --- | --- | --- |
| **Trunk alignment at take-off / support** | Trunk collapses forward; hips drop at board contact or during support | “Keep your trunk tall at take-off.” | “Lift your chest through support.” | “Drive up through the hips; avoid folding at the waist.” |
| **Shoulder angle at hand contact (support phase)** | Shoulders closed; elbows bend; hands contact too close to body | “Open the shoulders at hand contact.” | “Push long through the shoulders.” | “Hands firm, elbows straight; ‘reach then push’ off the box.” |
| **Hip lift & leg separation (flight/peak)** | Hip lift insufficient; legs separate late/uneven; ‘pike’ posture | “Lift the hips earlier.” | “Split the legs at the top, then close.” | “Think ‘up first, then split’; keep legs active and even.” |
| **Landing stability (final phase)** | Feet staggered; knee valgus; trunk sway; step on landing | “Stick the landing—quiet feet.” | “Land tall with knees over toes.” | “Absorb softly, stabilize, no extra steps; eyes forward.” |
| *(Optional)* **Approach rhythm / take-off timing** | Irregular steps; take-off too close/far; loss of speed control | “Keep a steady run-up rhythm.” | “Hit the board with a firm last step.” | “Maintain cadence; last step strong and controlled.” |

Table S1B. Delivery script (standardized wording)

| **Step** | **Standardized script** |
| --- | --- |
| 1. Start | “Let’s look at your last attempt.” *(start slow-motion replay, 3–5 s)* |
| 2. Cue 1 (primary) | “Key point: **[Cue A/B]**.” |
| 3. Cue 2 (secondary) | “Also: **[Cue A/B]**.” |
| 4. Action close | “Next trial, focus on these two points.” |

Table S1C. Cue-selection rule (to ensure consistency)

| **Rule** | **Specification** |
| --- | --- |
| Number of cues | Exactly **two** cues per KP episode (1 primary + 1 secondary). |
| Basis for selection | Selected based on the most salient execution errors observed in the immediately preceding trial video. |
| Prohibited content | No outcome statements (no KR), no evaluative scoring language (e.g., “7/10”), no comparative statements to peers. |
| Tone | Neutral, brief, action-oriented. |

Table S5. Descriptive statistics of psychological variables and performance outcomes

| **Variable** | **M** | **SD** |
| --- | --- | --- |
| Autonomous motivation | 4.98 | 1.03 |
| Metacognitive strategies | 3.00 | 0.83 |
| Acquisition mean score | 7.31 | 0.56 |
| Retention mean score | 7.40 | 0.55 |
| Transfer mean score | 7.36 | 0.57 |

**Note.** *M* = mean; *SD* = standard deviation.

Table S6. Fit indices for exploratory parallel mediation models

| Indicators | Retention Parallel | Transfer Parallel |
| --- | --- | --- |
| χ² | 412.50 | 637.14 |
| CFI | .839 | .734 |
| TLI | .676 | .596 |
| RMSEA | .108 | .175 |
| SRMR | .111 | .110 |

**Note.** These exploratory parallel mediation models specified autonomous motivation and metacognitive strategy use as concurrent mediators rather than sequential mediators. Across both outcomes, the parallel models showed substantially poorer overall fit than the theory-driven sequential models and therefore were not retained as the primary specification.
